# Supplementary material for: Viral Nucleases from Herpesviruses and Coronavirus in Recombination and Proofreading: Potential Targets for Antiviral Drug Discovery
Source: Viruses. 2022 Jul 16;14(7):1557. doi: 10.3390/v14071557 (PMC9324378; doi:10.3390/v14071557)
Supplement: Supplementary file 1 [file viruses-14-01557-s001.zip › viruses-1793275-supplementary.pdf]

Supplementary Materials

Viral Nucleases from Herpesviruses and Coronavirus in Recombination and Proofreading; Potential Targets for Antiviral Drug Discovery

Lee R. Wright, Dennis L. Wright and Sandra K. Weller

Table S1. Therapeutic agents used to treat HHV and HCoV infections

Drug Name	Approval	Indication	Compound Class	Target
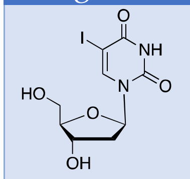 **Idoxuridine**	1962	HSV keratitis	nucleoside (deoxyuridine analog)	viral DNA polymerase
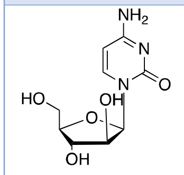 **Cytarabine**	1969	HSV (primarily used as an oncological agent)	nucleoside (cytosine analog)	viral DNA polymerase
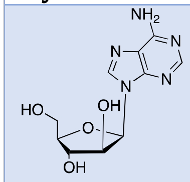 **Vidarabine**	1976	HSV, VZV (topical)	nucleoside (adenosine analog)	viral DNA polymerase
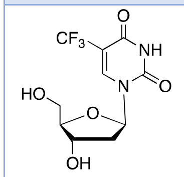 **Trifluridine**	1980	HSV keratitis	nucleoside (deoxythymidine analog)	viral DNA polymerase
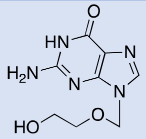 **Acyclovir**	1982	HSV, VZV	nucleoside (guanosine analog)	viral DNA polymerase

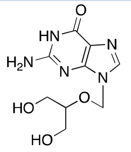 **Ganciclovir**	1988	CMV, HSV keratitis	nucleoside (guanosine analog)	viral DNA polymerase
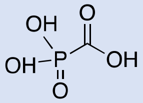 **Foscarnet**	1991	HSV, CMV	Phosphonate (pyrophosphate mimic)	viral DNA polymerase
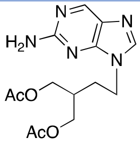 **Famciclovir**	1994	Herpes zoster	penciclovir diacetate ester prodrug	viral DNA polymerase
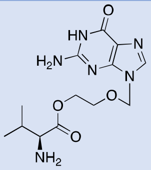 **Valaciclovir**	1995	HSV (general)	acyclovir valine ester prodrug	viral DNA polymerase
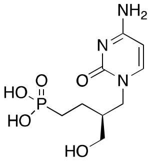 **Cidofovir**	1996	CMV retinitis	nucleotide (cytidine analog)	viral DNA polymerase
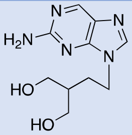 **Penciclovir**	1996	Herpes labialis (topical)	nucleoside (guanosine analog)	viral DNA polymerase
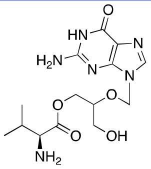 **Valganciclo vir**	2001	CMV	ganciclovir valine ester prodrug	viral DNA polymerase
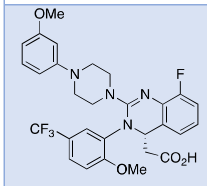 **Letermovir**	2017	CMV	allosteric inhibitor	CMV terminase

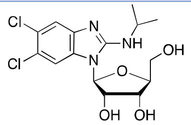<br>**Maribavir**	2021	CMV (refractory)	kinase inhibitor	CMV pUL97
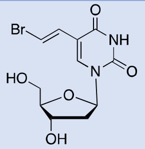<br>**Brivudine**	Europe only	Herpes zoster	nucleoside (deoxythymidine analog)	viral DNA polymerase
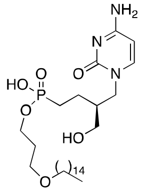<br>**Brincidofovir**	Investigational (approved 2021 for smallpox)	broad HHV	cidofovir lipid conjugated prodrug	viral DNA polymerase
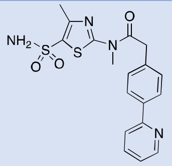<br>**Pritelivir**	Investigational	HSV	allosteric inhibitor	HSV helicase-primase complex
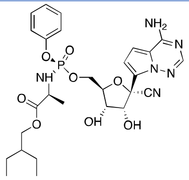<br>**Remdesivir**	2020	SARS-CoV-2	nucleotide prodrug	RNA-dependent RNA-polymerase
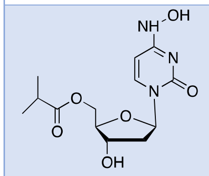<br>**Molnupiravir**	EUA (2021)	SARS-CoV-2	nucleoside prodrug (cytidine analog)	RNA-dependent RNA-polymerase
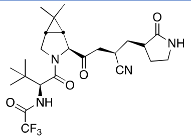<br>**Nirmatrelvir**	EUA (2021, in combination with ritonavir)	SARS-CoV-2	protease inhibitor	Viral C3-like-protease
